# Relational coordination among healthcare professionals in acute care: a mixed-methods study of tasks involving prehospital assessment units

**DOI:** 10.1186/s12913-026-14103-2

**Published:** 2026-02-02

**Authors:** Helle Mätzke Rasmussen, Anders Løkke, Peter Biesenbach, Annmarie Lassen, Anne Friesgaard Christensen, Eva Hoffmann, Søren Mikkelsen, Mette Elkjaer

**Affiliations:** 1 Research Center for Integrated Healthcare, Region of Southern Denmark, University Hospital of Southern Denmark, Kresten Philipsens Vej, Aabenraa, Denmark; 2 Department of Regional Health Research, University of Southern Denmark, Odense, Denmark; 3 Department of Medicine, Lillebaelt Hospital, Vejle, Denmark; 4 Department of Emergency Medicine, Esbjerg Hospital, Esbjerg, Denmark; 5 Department of Emergency Medicine, Odense University Hospital, Odense, Denmark; 6 Institute of Clinical Research, University of Southern Denmark, Odense, Denmark; 7 Department of Internal Medicine, Lillebaelt Hospital, Kolding - University Hospital of Southern Denmark, Kolding, Denmark; 8 University College South Denmark, Aabenraa, Denmark; 9 The Prehospital Research Unit, Department of Regional Health Research, University of Southern Denmark, Odense, Denmark

**Keywords:** Relational coordination, Emergency medical services, Prehospital assessment unit, Non-conveyance, Interprofessional collaboration

## Abstract

**Background:**

Prehospital Assessment Units were recently introduced to emergency medical services in Denmark. These units are designed to assess, triage, and treat patients on-site, and either release, refer to alternative care pathways, or arrange hospital conveyance. The initiative has increased the need for effective collaboration among healthcare professionals in acute care services, including paramedics, nurses, and physicians in emergency medical services, municipalities, emergency departments, and general practice. This study investigates Relational Coordination among healthcare professionals involved in this task for patients triaged by the Prehospital Assessment Units in the Region of Southern Denmark.

**Methods:**

A mixed-methods study, inspired by a convergent parallel design, was conducted based on the framework described in the theory of Relational Coordination. This theory highlights the importance of Communication (Frequent Communication, Timely Communication, Accurate Communication, and Problem-Solving Nature of Communication) and Relationships (Shared Goals, Shared Knowledge, and Mutual Respect). Data were collected between April and August 2024 via online questionnaires and in June 2024 through focus groups.

**Results:**

Questionnaire responses from 138 healthcare professionals were analysed, and 18 participated in the focus groups. Most collaborative ties were classified as moderate and strong. However, weaker ties were identified in Timely Communication and Frequent Communication, and Shared Knowledge, highlighting challenges in both dimensions of Relational Coordination. The qualitative analysis produced four themes: 1) *Insufficient communication * due to low quality or lack of written communication, 2) *Navigating care through phone calls *to share information, 3) *Multiple objectives with hindered observers*, driven by the coexistence of different objectives of the unit and limited insight into the whole care pathway, and 4) *Interprofessional tensions in acute care collaboration*, based on challenges regarding the competencies, incentives, roles and responsibilities required to achieve the best care pathway for each patient.

**Conclusion:**

The collaboration among healthcare professionals for patients triaged by the Prehospital Assessment Unit was generally classified as moderate to strong. However, critical challenges were identified. Optimising task assignments, enabling municipal correspondence, and addressing discrepancies in objectives, competencies, and roles may improve collaboration. These findings point to broader lessons for strengthening prehospital and inter-organisational coordination internationally, where challenges of information flow, role clarity, and mandate alignment are common.

**Supplementary Information:**

The online version contains supplementary material available at 10.1186/s12913-026-14103-2.

## Introduction

Globally, ambulance services have undergone substantial transformations, evolving from transport-focused services responsible for conveying patients from the scene to hospitals into sophisticated Emergency Medical Services (EMSs) providing prehospital triage and acute care [1]. Prehospital Assessment Units (PAUs) have recently been integrated into EMS in Denmark. These specialised units are designed to assess, triage, and treat patients on-site and, subsequently, either release, refer to alternative care pathways, or arrange hospital conveyance. Research indicates that this new type of service provides a valuable alternative to existing acute care services [2], without compromising safety [3, 4].

PAUs provide an alternative to other acute care services by serving the same patient populations as paramedics, nurses, and physicians in EMSs, Emergency Departments (EDs), municipalities, and general practice settings. These healthcare professionals (HCPs) can refer patients to the unit, and the unit can request other services to offer alternatives to hospital conveyance. Thus, the services provided by PAUs add to the complexity of acute care [2, 5] and heighten the need for effective collaboration, which has been suggested as important for creating appropriate care pathways [2] and in understanding variations in non-conveyance between ambulance services [5]. Non-conveyance is the decision not to transport patients to the hospital after prehospital triage and is a key outcome for PAUs.

Recent studies have emphasised that collaboration in acute and transitional care is not merely procedural but actively negotiated across organisational boundaries, shaped by diverging goals, incentives, and role expectations [6]. This perspective is particularly relevant for PAUs, which operate at the intersection of EMS, EDs, municipalities, and general practice. Ambulance clinicians have also highlighted the challenges and opportunities of interprofessional collaboration in prehospital care for older patients with complex needs, underscoring the importance of shared understanding and coordinated decision-making [7].

The importance of Relationships and Communication in achieving effective collaboration has been highlighted in the research-based framework Relational Coordination (RC) theory [8, 9]. The theory posits that strengthening RC leads to better stakeholder outcomes [10]. Furthermore, research suggests that integrating new types of services is more effective when RC levels are moderate to high [11].

The RC Survey examines Relationships and Communication between roles involved in a task process [8, 12]. To complement survey data, qualitative approaches can provide deeper insight into collaboration by capturing participants’ experiences and perspectives. Reflexive Thematic Analysis (RTA), as described by Braun and Clarke [13], is grounded in a social constructivist orientation, assuming that meaning is not passively discovered but actively constructed through interaction between participants and interpreted by the researcher. Together, these approaches capture both measurable patterns of RC and the nuanced experiences of HCPs. In the acute care setting, this framework has previously been utilised to examine collaboration in an ED [10], a primary care setting [14], and an emergency medical dispatch centre [15]. Thus, providing a robust framework for assessing collaborations across roles due to its feasibility and practical applicability in real-world settings [10, 16].

As part of a broader quality assurance project evaluating the PAU within acute care services in the Region of Southern Denmark, the specific aim of this study was to investigate RC among HCPs involved in the task process for patients triaged by the PAU.

## Methods

### Study design

**Fig. 1:**
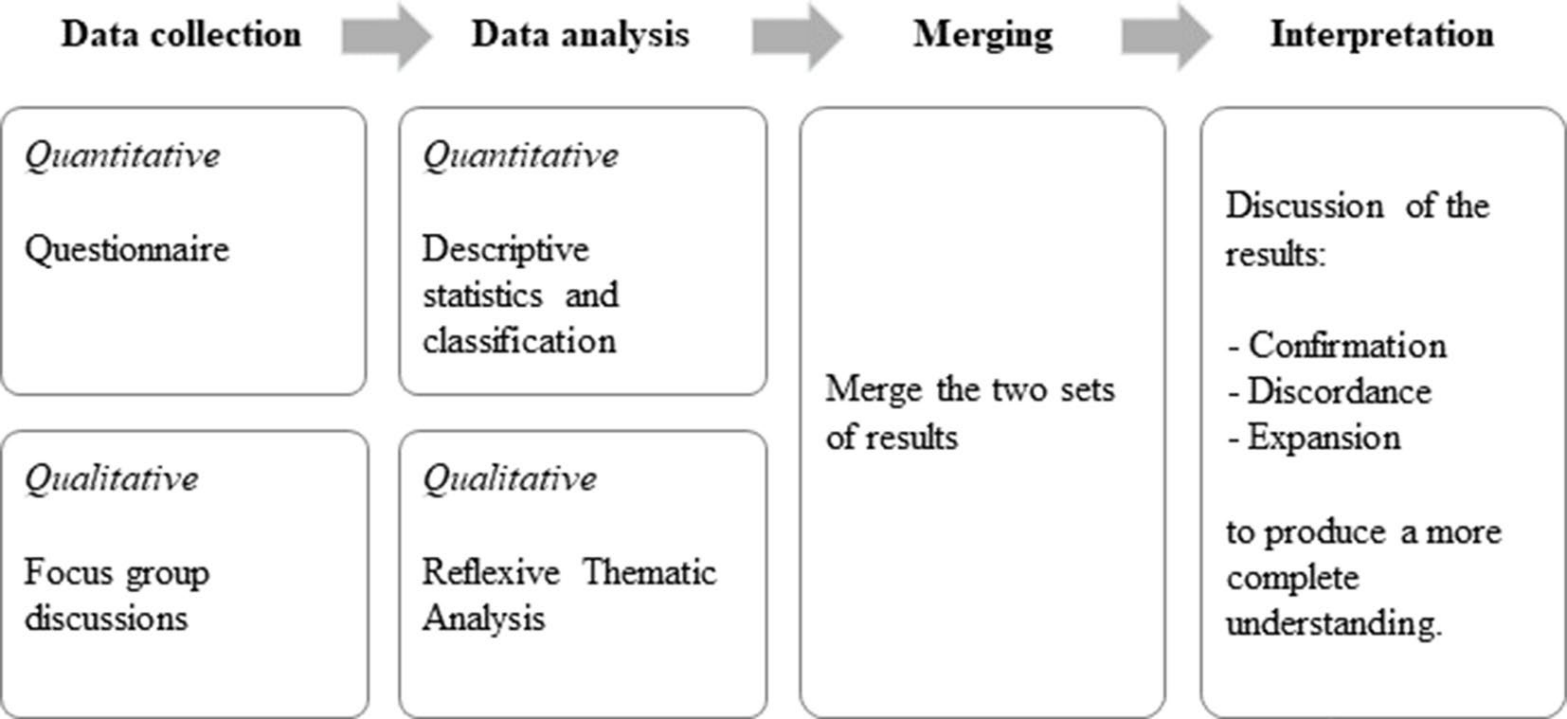
Study design. Figure legend: Outline of the study design inspired by the convergent parallel design, including key phases and method applied in data collection and analysis

We designed a mixed-methods study inspired by a convergent parallel design [17, 18], in which quantitative and qualitative stands were implemented concurrently. Specifically, data were collected from questionnaires and focus groups during the same timeframe, April to August 2024 (Fig. 1). The study was informed by RC theory, which emphasises the interdependence between roles and the importance of effective collaboration for task integration. This theory provided the foundation for both the design of the RC Survey and the initial deductive coding applied in the qualitative analysis [8, 9]. The reporting of this study follows the Good Reporting of A Mixed Methods Study in Health Services Research (GRAMMS) checklist [19].

### Study setting

Denmark has a tax-funded healthcare system with three levels. The state regulates and supervises five health regions. These regions manage hospitals including EDs, EMSs, and general practitioners (GPs), whilst municipalities manage long-term care and acute home care. Responsibility for acute care services is divided between EDs, EMSs, GPs, and municipal acute care teams [20]. Patients experiencing potential life-threatening emergencies or accidents are primarily cared for by the EMSs and EDs. The first line of care for patients experiencing exacerbations of pre-existing conditions or new acute medical issues is provided by GPs and municipal acute care facilities, while the second line of care is handled by EDs, with the EMSs responsible for conveyance [20]. Three PAUs were established in 2023 as an add-on to the EMS in the Region of Southern Denmark. The units can care for all the aforementioned patients, making patients eligible for at least two concurrent services.

The PAU is a vehicle equipped for comprehensive patient assessment, including an ultrasound scanner and blood analysis equipment, but does not include a stretcher or examination room, and cannot transport patients. It is activated by the Emergency Medical Dispatch Centre in response to emergency calls or referrals from an HCP, typically from the ED. Each unit is staffed by a paramedic who, in close collaboration with an ED physician, performs prehospital triage and determines the best care pathway, including whether treatment can safely be provided at home.

### Participants

**Fig. 2:**
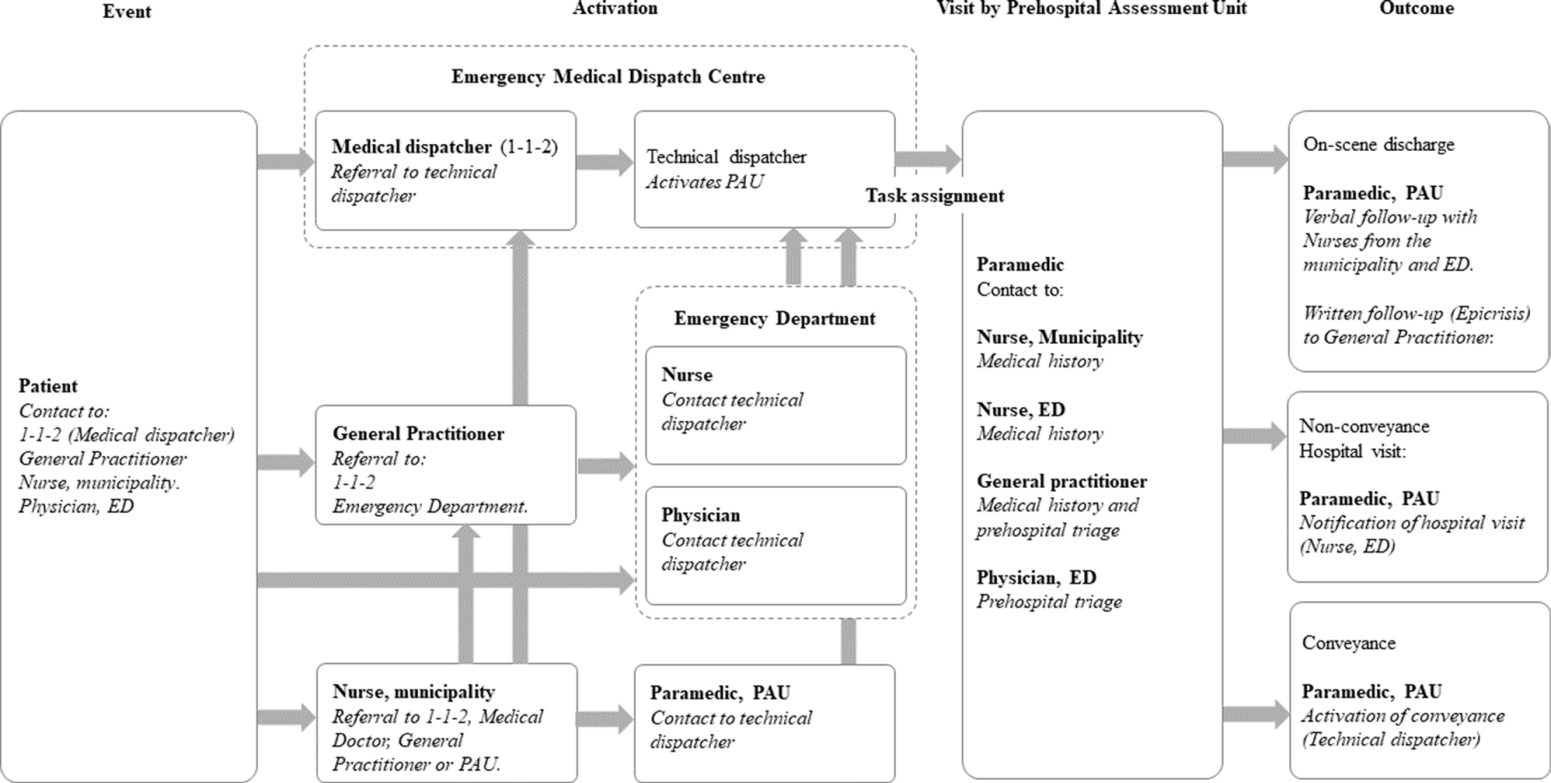
Task process. Figure legend: Task process for patients receiving prehospital triage by Prehospital Assessment Unit in the Region of Southern Denmark. The process begins with an event, followed by activations of and a visit by the unit, and concludes with one of the defined outcomes. Abbreviations: ED: Emergency Department, PAU: Prehospital Assessment unit

A description of the task process (Fig. 2) was used to identify HCPs holding specific roles in the care pathways for patients receiving prehospital triage by a PAU. In the involved organisations, six roles were identified:

1. Coordinating nurses, ED
2. Physicians specialised in emergency medicine, ED
3. Nurses from acute care teams and home care service, Municipalities
4. GPs
5. Paramedics manning the PAU, EMS
6. Medical dispatchers staffing the Emergency Medical Dispatch Centre, EMS

All organisations accepted the invitation to participate and provided contact information for individuals holding the identified roles in their organisation. An introductory e-mail with a link to the questionnaire was sent to 339 individuals, while 108 GPs were invited via a postal letter. All respondents were invited to participate in the focus groups. GPs were invited through the GP Consultant Scheme at the EDs. In composing each of the three focus groups, we aimed to ensure representation from all six identified roles in the care pathway, with participants affiliated to the same naturally occurring geographical groups defined by the catchment area of an ED. This approach reflects recommendations in focus group methodology to combine heterogeneity across roles with the use of naturally occurring groups, thereby facilitating inter-professional discussion while grounding participants in shared organisational contexts [21].

### Data collection

#### Quantitative data: online questionnaire

Quantitative data were collected via an online questionnaire using web-based SurveyXact from April to August 2024. Data on (1) sociodemographic characteristics and knowledge about the PAU; and (2) RC, were retrieved and analysed in the current study.

Sociodemographic data on role, experience in current role, and place of work were collected. Furthermore, the participant’s level of experience with the PAU was rated on a 5-point response scale from 1 to 5.

RC was assessed across roles involved in the task process using a Danish-translated version of the RC Survey [22]. The survey comprised seven questions to measure the two dimensions of RC: Communication (Frequent Communication, Timely Communication, Accurate Communication, and Problem-Solving Nature of Communication) and Relationships (Shared Goals, Shared Knowledge, and Mutual Respect). It assesses the strengths and weaknesses of Relationships and Communication as ties between roles [9].

The questions are answered on a 3 to 5-point scale with the option to answer ‘not relevant’ for each role. Psychometric properties for the original English version have been evaluated [12], while the Danish version has been translated using a cross-cultural adaptation process [22].

#### Qualitative data: focus groups

Three focus groups were held in June 2024. The method was selected to enable in-depth exploration of RC among HCPs and to facilitate inter-professional interactions, thereby revealing potentially differing experiences and opinions from the various roles involved. Focus groups are particularly suited to health service research as they enable exploration of experiences and reveal how differing perspectives are articulated and negotiated through group interaction [21]. The focus groups were conducted face-to-face and lasted about 2 h. The Focus Group Discussion Guide (Additional File 1) was designed based on constructs from the quality assurance project. We aimed to explore participants’ experiences with PAU and perspectives on care pathways using fictive cases to facilitate discussion across roles and support inter-professional exchange.

Author HMR coordinated the focus groups and served as a secretary during the sessions, taking field notes, tracking time, and presenting the cases. Author EH moderated the discussions, applying strategies to encourage contributions from all participants and to manage potential hierarchical dynamics, in line with methodological recommendations for focus group research [21]. Both authors were middle-aged females with more than 20 years of experience as HCPs but limited experience in prehospital and acute care settings, which fostered a genuine curiosity about the participants’ experiences with the PAU.

The focus groups were audio-recorded using an encrypted device. Author HMR transcribed the focus groups verbatim, anonymised the transcripts by assigning participant numbers, and removed identifying information; transcripts were not shared with the participants. All quotations were anonymised using role-level attribution (e.g., “Paramedic”) and stripped of site-specific identifiers to preserve confidentiality while retaining contextual meaning, in line with RTA guidance.

### Data analysis

RC theory served as the overarching analytical framework for both the quantitative and qualitative components of the study. RC defines coordination as a mutually reinforcing process of Communication and Relationships aimed at task integration [8, 9]. Within RC theory, collaboration between two roles is conceptualised as a ‘tie’, the quality of which is essential for effective coordination. The framework identifies seven dimensions, grouped into two categories:

- Communication: Frequent Communication, Timely Communication, Accurate Communication, and Problem-Solving Nature of Communication
- Relationships: Shared Goals, Shared Knowledge, and Mutual Respect.

#### Quantitative analysis

Descriptive statistics were calculated to describe the participants. Based on the RC survey data, we calculated the means and standard deviations for the seven individual RC dimensions. In addition, mean and standard deviation values were computed for the composite Communication score (derived from the four communication dimensions within RC), and the composite Relationships score (based on the three relationship dimensions within RC). Finally, the overall RC mean score and its standard deviation were calculated as the average of all seven dimensions. Scores were initially calculated at the individual level and then aggregated to the role level by averaging across all respondents within each of the six roles, in line with instructions from the developers of the RC survey [9]. ‘Not relevant’ responses to the RC survey were omitted from further analysis. Ties were classified by type and score according to Gittell et al. [9]. Ties within roles were categorised as weak (< 4.1), moderate (4.1–4.6), or strong (> 4.6). Ties between roles were classified as weak (< 3.5), moderate (3.5-4.0), or strong (> 4.0). Ties between organisations were classified as weak (< 3.0), moderate (3.0-3.5), or strong (> 3.5). The difference in thresholds was justified by expected relational density. RC is typically stronger within roles, weaker across roles, and weakest across organisations, reflecting increasing structural and relational boundaries [9, 10].

Proportions of weak, moderate, and strong ties were calculated using the number of valid responses within each role as the denominator, and percentages were used to enable comparison across roles and dimensions. All statistical analyses were performed using Stata/BE (StataCorp).

#### Qualitative analysis

The qualitative analysis was conducted from a constructivist perspective. Author HMR led the analysis following Braun and Clarke’s recommendations for RTA [13] of the complete dataset from the focus groups. The analysis was undertaken from a contextualist epistemological stance, recognising participants’ accounts as meaningful while also situating them within the organisational conditions shaping acute care collaboration. Reflexive thematic analysis was chosen because it aligns with this constructivist stance, assuming that meaning is actively constructed through participants’ interactions and interpreted by the researcher. The constructivist perspective, contextualist epistemological stance, and the professional background of the author, HMR (an experienced healthcare practitioner, but relatively new to prehospital care) fostered genuine curiosity and reflexive thinking. This reflexive positioning shaped the analysis process, including theme development and interpretation, by encouraging sensitivity to inter-professional differences and the recognition of shared perspectives, consistent with Braun and Clarke’s recommendations for RTA [13].

Data were managed in NVivo (Lumivero) and coded using a combined deductive–inductive approach. Deductive codes were informed by the seven dimensions of RC theory [8, 9], while inductive codes were developed at both semantic and latent levels to capture participants’ diverse and sometimes contrasting perspectives. RC constructs were used as sensitising concepts rather than fixed categories, and the reasoning process integrated deductive, inductive, and abductive elements. Abductive reasoning was particularly important when interpreting surprising or ambiguous accounts. For example, frequent references to phone calls were not only coded as communication but abductively interpreted as inter-organisational workarounds through which professionals sought to secure timely information exchange. This iterative movement between data, RC theory, and organisational context yielded novel insights.

Codes were collated into three preliminary themes, which were subsequently refined into four final themes through iterative discussions with the co-authors. Reflexivity was supported through memoing, supervisory input, and team dialogue (EH, ME, ALo), ensuring nuanced interpretation and consideration of alternative explanations. The findings were not shared with participants.

A detailed overview of the six analytic phases, including examples of how codes were developed and themes refined, is presented in Table 1.

**Table 1:** Reflexive thematic analysis: phases and activities

Phase, heading and description
**1 Familiarising with the dataset**: Transcripts were printed, read, and the audio recordings were listened to in full, while noting reflections by HMR, while EH and ME reviewed selected parts of the transcripts to familiarize themselves with the data. Early observations included both descriptive and interpretive notes on Communication and Relationships. This immersion sensitised the analysis to potential links with RC theory and highlighted that participants voiced RC dimensions in diverse and sometimes contrasting ways. The process was undertaken from a contextualist stance, recognising both participants’ accounts and the organisational conditions shaping them.
**2 Coding**: Building on these observations, data were manually coded using a combined deductive–inductive approach by HMR. Deductive codes were drawn from RC theory dimensions (Frequent Communication, Timely Communication, Accurate Communication, Problem-Solving Nature of Communication, Shared Goals, Shared Knowledge, and Mutual Respect). To capture the variation identified during familiarisation, inductive codes were developed at both semantic and latent levels (e.g., *Backstapper*, *Our hands*,* eyes and ears*, *Please ask me*). In total, 32 codes were generated, each linked to one of the RC dimensions but shaped by participants’ own accounts. RC constructs were used as sensitising concepts rather than fixed categories, and the reasoning process combined deductive and inductive codes to remain open to novel insights. EH and ME provided feedback on the codes.
**3 Generating initial themes**: Codes were collated into three preliminary themes by HMR. For example, *Not obligated beyond one’s scope of responsibilities* grouped codes such as *Backstapper*, *Different levels of competencies*, *Hierarchical system*, and *Passing the ball*. These reflected both theoretical alignment and emergent data-driven insights. Themes were developed at different levels of abstraction, often leaning toward the latent side of analysis. Some themes captured participants’ explicit accounts (e.g., missing correspondence messages), others required interpretation of underlying assumptions and tensions (e.g., when discussing hierarchy).
**4 Developing and reviewing themes**: Themes were reviewed against coded extracts and the dataset by HMR. Through iterative discussions between co-authors (HMR, EH and ME), themes were refined and divided where needed. For example, *Insufficient written communication drives phone calls* was split into *Insufficient written communication* and *Navigating care through phone calls*, reflecting distinct patterned meanings: (a) inadequate documentation and (b) active use of phone calls before, during, and after visits. Reflexivity was supported through co-author discussions, and consideration of alternative interpretations during this phase.
**5 Refining**,** defining and naming themes**: The four themes were checked across the dataset to ensure coherence and distinctiveness by HMR. Each was defined around a central organising concept, with names chosen to reflect the appropriate level of abstraction. Co-author discussions (EH, ALo and ME) supported reflexive consideration and nuanced interpretation. Themes were evaluated against quality criteria of coherence, credibility, and richness to ensure they captured patterned meanings across the dataset.
**6 Writing up**: Final themes were written up to present a coherent analytic narrative. This included illustrative extracts, links to RC theory, and positioning within the broader literature. The write-up aimed to balance accessibility with analytic depth, demonstrating how themes were grounded in the data while interpreted through a reflexive, theory-informed lens. All authors provided feedback on the final themes.

This table provides a transparent overview of the six phases of reflexive thematic Analysis, showing how the process moved from immersion in the data to the generation and refinement of themes. The analysis was not linear but iterative, with movement back and forth between phases as ideas were developed, tested, and refined. The descriptions highlight the reflexive nature of the process, the integration of deductive, inductive, and abductive reasoning, and its grounding in RC theory. Abbreviations: RC, Relational Coordination

### Merging and interpretation

Quantitative results and qualitative findings were placed in a matrix grouped by three concepts: RC, Communication, and Relationships, for a visual comparison by author HMR. Lines were drawn to mark where data converged, diverged, or related to quantitative results and other qualitative findings and to guide the integration process. From these comparisons, meta-inferences were generated by identifying convergent patterns across strands and linking them to actionable implications, which were then elaborated in the Discussion. The process and results were discussed with ME and ALo.

### Ethical considerations

The project was classified as a quality assurance initiative and approved by the Prehospital Medical Manager in the Region of Southern Denmark. In accordance with Danish legislation, the study was exempt from formal ethical review under the Danish Medical Research Involving Human Subjects Act (§ 14).

Questionnaire respondents received written information about the study as part of the survey materials, and their consent was deemed by completing and returning the questionnaire. Focus group participants were provided with both written and oral information and gave explicit written consent prior to participation, with the option to withdraw at any stage before the qualitative material was analysed.

Questionnaire data were collected in anonymised form, and no sensitive personal details were obtained. Focus group discussions were recorded on an encrypted device and stored securely on a regionally approved SharePoint platform with access restricted to the research team. Confidentiality and anonymity of all participants were safeguarded throughout.

The study adhered to the principles of the Declaration of Helsinki [23]. Data handling complied with the General Data Protection Regulation (GDPR), and the processing of personal data was registered with and approved by the Region of Southern Denmark (journal no. 24/18181) in accordance with Article 30 of the EU GDPR.

## Results and findings

### Characteristics of participants

A total of 231 HCPs responded to the questionnaire (51.7% response rate). Fifty-four participants were excluded for lack of PAU experience, and 39 for incomplete RC responses (leaving the questionnaire before or during the RC Survey), giving a total of 94 exclusions. A total of 138 valid responses were included in the analysis. Thirty-four HCPs indicated interest in participating in the focus groups in the questionnaire and were subsequently invited to one of the three sessions; 22 accepted. Eighteen HCPs participated (9, 4, and 5 participants in each group). Twelve invitees did not reply to the invitation or were unable to attend at the scheduled times and therefore did not participate, while four cancelled immediately prior to the focus groups due to unexpected personal circumstances and clinical duties. Participants included paramedics, nurses, and physicians in EMSs, EDs, municipalities, and general practice settings. Participation reflected the availability of staff from the six roles across organisations, and in practice, nurses and paramedics were more frequently represented than medical doctors. Their characteristics are detailed in Table 2.

**Table 2:** Characteristics of participants

	Questionnaire			Focus group
	All respondents	Analysis		Analysis
	n (%)	n (%)		n (%)
Total	231 (100.0)	138 (100.0)		18 (100.0)
**Role**				
Nurse, ED	38 (16.5)	20 (14.5)		1 (5.6)
Physician, ED	59 (25.5)	44 (31.9)		3 (16.7)
Nurse, Municipality	85 (36.8)	38 (27.5)		8 (44.4)
General Practitioner	11 (4.8)	5 (3.6)		1 (5.6)
Paramedic on PAU	21 (9.1)	18 (13.0)		5 (27.8)
Medical dispatcher	17 (7.4)	13 (9.4)		-
**Experience in current role**				
0–5 year	130 (56.3)	74 (53.6)		7 (38.9)
6–10 years	35 (15.2)	22 (15.9)		2 (11.1)
> 10 years	66 (28.6)	42 (30.4)		9 (50.0)
**Experience with PAU**				
No experience	54 (23.4)	-		-
1–10 patient contacts	60 (26.0)	44 (31.9)		8 (44.4)
≥ 10 patient contacts	117 (50.7)	94 (68.1)		10 (55.6)

Participants responding to the questionnaire and attending focus groups. Categorized by role, years in current position, and experience with the PAU. The questionnaire columns show all respondents and those included in the analysis based on relevant PAU experience and completeness of responses. Data shown as counts and percentages to enable comparison; percentages based on small denominators should be interpreted with caution

Abbreviations: ED, Emergency Department; PAU, Prehospital Assessment Unit﻿

The characteristics of participants were not directly included in the quantitative or qualitative analyses but served as contextual background to inform the interpretation of findings, ensuring that results were understood in light of the organisational roles and group composition.

### Quantitative results and qualitative findings

The quantitative examination of RC revealed that most of the ties were classified as moderate or strong. However, it also revealed that seven of the 36 ties were classified as weak, as outlined in the matrix diagram in Table 3. Four of these weak ties were reported by medical dispatchers. Additionally, weak ties were identified between ED physicians themselves, between paramedics and GPs, and finally, the tie between GPs themselves was classified as weak.

**Table 3:** Relational Coordination

Relational Coordination	Reported with these roles					
Reported by these roles Mean (SD) *N* = 138	Nurse, ED	Physician, ED	Nurse, Municipality	General Practitioner	Paramedic on PAU	Medical dispatcher
Nurse, ED	4.3 (0.7)	*4.1 (0.6)*	3.5 (0.8)	3.4 (0.8)	*4.4 (0.8)*	*3.8 (1.0)*
Physician, ED	*4.2 (0.8)*	4.3 (0.8)	3.3 (0.9)	**2.8 (0.9)**	*4.6 (0.5)*	3.1 (1.2)
Nurse, Municipality	3.2 (1.1)	3.5 (1.0)	4.4 (0.8)	3.3 (0.9)	*3.9 (0.7)*	3.0 (0.9)
General Practitioner	*3.9 (0.6)*	3.2 (0.7)	*3.8 (0.8)*	**3.3 (1.5)**	*4.1 (0.6)*	3.2 (1.3)
Paramedic, PAU	*4.1 (0.6)*	*4.5 (0.3)*	*3.7 (0.9)*	**2.7 (0.5)**	*4.7 (0.4)*	*3.5 (0.7)*
Medical dispatcher	**2.6 (1.3)**	**2.6 (0.9)**	**2.6 (1.1)**	**2.5 (1.1)**	3.4 (1.0)	4.1 (1.1)

Overall mean Relational Coordination scores within and between roles. Bold values indicate ties classified as weak; italicised values indicate ties classified as strong. Abbreviations: ED, Emergency Department; PAU, Prehospital Assessment Unit; SD, standard deviation

When exploring the RC between the roles, the analysis produced four intersecting themes: *(1) Insufficient communication*,* (2) Navigating care through phone calls*,* (3) Multiple objectives with hindered observers*, and *(4) Interprofessional tensions in acute care collaboration.*

The themes illustrate different aspects of Relationships and Communication, shaping the overall RC. While participants viewed PAUs as beneficial for patients, professionals, and society, the themes highlight challenges that may affect the task processes and patient outcomes.

#### Communication

Quantitative data on Communication is presented in Table 4. Of the 144 ties, 42 (29%) were classified as weak. In Frequent Communication, the majority of ties were classified as weak. Physicians from EDs reported weak ties with all roles except paramedics; nurses from municipalities reported weak ties with nurses and physicians from EDs and the medical dispatcher; GPs reported weak ties with all roles except paramedics and the medical dispatcher; paramedics reported weak ties with GPs and the medical dispatchers.

**Table 4:** Communication dimension of Relational Coordination

Reported by these roles Mean (SD) *N* = 138	Nurse, ED	Physician, ED	Nurse, Municipality	General Practitioner	Paramedic on PAU	Medical dispatcher
**Frequent Communication**						
Nurse, ED	*3.8 (1.1)*	3.6 (1.1)	3.1 (1.1)	3.3 (1.3)	*4.1 (1.2)*	3.4 (1.4)
Physician, ED	**3.4 (1.1)**	**3.4 (1.5)**	**2.5 (1.1)**	**1.8 (0.8)**	*4.3 (0.9)*	**2.1 (1.3)**
Nurse, Municipality	**2.3 (0.9)**	**2.9 (1.2)**	4.1 (1.1)	3.0 (1.1)	*3.9 (0.8)*	**2.3 (0.9)**
General Practitioner	**2.8 (1.5)**	**2.0 (0.7)**	**2.8 (1.6)**	**1.5 (0.6)**	*3.6 (1.1)*	3.2 (1.5)
Paramedic, PAU	*3.6 (1.0)*	*4.4 (0.6)*	3.3 (1.1)	**2.2 (0.5)**	4.0 (1.3)	**2.7 (1.1)**
Medical dispatcher	**1.8 (1.0)**	**1.6 (1.1)**	**2.0 (1.3)**	**1.9 (1.3)**	**2.6 (0.8)**	**3.9 (1.4)**
**Timely Communication**						
Nurse, ED	*4.0 (1.0)*	3.8 (1.1)	3.1 (1.0)	3.4 (1.1)	*4.4 (0.9)*	*3.7 (1.3)*
Physician, ED	*4.1 (1.2)*	**3.9 (1.3)**	3.3 (1.5)	**2.4 (1.3)**	*4.6 (0.7)*	**2.9 (1.6)**
Nurse, Municipality	3.0 (1.2)	3.1 (1.3)	4.4 (0.9)	3.2 (1.1)	*3.9 (1.0)*	**2.7 (1.0)**
General Practitioner	3.3 (1.3)	**2.3 (0.5)**	3.4 (1.5)	**1.7 (0.6)**	*3.6 (1.5)*	3.0 (1.4)
Paramedic, PAU	*3.8 (0.9)*	*4.5 (0.5)*	3.4 (1.0)	**2.4 (0.6)**	4.5 (0.8)	**2.9 (1.2)**
Medical dispatcher	**2.3 (1.9)**	**1.7 (1.3)**	**2.0 (1.4)**	**1.9 (1.2)**	**2.8 (1.3)**	**3.8 (1.4)**
**Accurate Communication**						
Nurse, ED	4.1 (0.9)	*4.1 (0.7)*	3.4 (0.8)	*3.7 (0.07)*	*4.5 (0.6)*	*3.9 (0.9)*
Physician, ED	*4.4 (0.8)*	4.4 (1.0)	*3.6 (1.2)*	3.4 (1.3)	*4.5 (0.7)*	3.5 (1.5)
Nurse, Municipality	*3.8 (0.9)*	*3.7 (1.2)*	4.5 (0.5)	*3.7 (0.7)*	*4.1 (0.9)*	3.5 (1.1)
General Practitioner	*4.0 (0.7)*	3.5 (1.0)	*4.0 (0.7*)	**4.0 (0.0)**	*4.4 (0.5)*	*4.3 (0.5)*
Paramedic, PAU	*4.2 (0.4)*	*4.6 (0.5)*	*3.7 (1.1)*	**2.8 (0.9)**	*4.6 (0.7)*	3.5 (0.9)
Medical dispatcher	**2.8 (2.0)**	**2.3 (1.5)**	**2.5 (1.2)**	**2.6 (1.5)**	**3.3 (1.3)**	**3.9 (0.9)**
**Problem-Solving Nature of Communication**						
Nurse, ED	4.6 (0.8)	*4.4 (0.6)*	*4.3 (1.0)*	3.3 (1.0)	*4.7 (0.8)*	*4.1 (1.0)*
Physician, ED	*4.7 (0.7)*	*4.8 (0.6)*	*4.3 (1.0)*	*3.9 (1.3)*	*4.8 (0.6)*	*4.5 (0.9)*
Nurse, Municipality	*4.6 (0.8)*	*4.5 (0.9)*	*4.9 (0.4)*	*3.8 (1.2)*	*4.4 (0.9)*	*4.0 (1.0)*
General Practitioner	*4.6 (0.9)*	*4.2 (1.1)*	*4.2 (1.1)*	4.5 (1.0)	*4.6 (0.9)*	*4.0 (1.2)*
Paramedic, PAU	*4.6 (0.8)*	*4.9 (0.5)*	*3.9 (1.2)*	3.1 (1.1)	*4.7 (0.8)*	4.0 (1.3)
Medical dispatcher	*3.7 (1.2)*	3.0 (0.0)	3.4 (0.9)	3.5 (1.0)	3.8 (1.5)	*5.0 (0.0)*

Mean score for the four questions representing the communication dimension of Relational Coordination within and between roles. Bold values indicate ties classified as weak; italicised values indicate ties classified as strong. Abbreviations: ED, Emergency Department; PAU, Prehospital Assessment Unit; SD, standard deviation

In the Timely Communication dimension, 13 (33%) ties were classified as weak. Physicians from EDs reported weak ties with physicians from EDs and GPs and the medical dispatchers; nurses from municipalities reported weak ties with the medical dispatcher; GPs reported weak ties with physicians from EDs and GPs; paramedics reported weak ties with GPs and the medical dispatchers.

In Frequent Communication, Timely Communication, and Accurate Communication, medical dispatchers reported weak ties with all roles.

The theme *Insufficient written communication* describes how low-quality, insufficient written communication between the paramedic manning the PAU and other roles is experienced, impacting task execution and potentially the outcome.

Participants highlighted how insufficient written communication affected their task execution before and after the visit, when a patient was released at the scene. This theme highlights how gaps in written communication can pose risks to patients and lead to inefficiencies.

A task assignment is sent to the PAU before the visit, as outlined in Fig. 2. The task assignment is given to a technical dispatcher by the medical dispatcher or the HCP who activates the unit. Sometimes, the assignment is written simply as the word “fluid”. When an assignment lacks sufficient detail, paramedics make phone calls, sometimes to several colleagues, to obtain more detailed information. This highlighted the potential risk to patient safety:

*I’ve experienced once*,* that the medical dispatcher had discussed with the technical dispatcher that the PAU might be the best unit to activate because the patient may be dehydrated. Then the technical dispatcher wrote “fluid” … that task assignment is right on the edge of having a non-healthcare professional essentially prescribing fluids.* (Paramedic)

When PAU releases a patient, the paramedic sends a notification to the GP from the prehospital patient record. If the paramedic needs to share information with HCPs in a municipality, it can be done in two ways: either in writing, relayed by an ED employee, or by phone contact with an employee in the municipality. Thus, a large unmet need exists for a formalised transfer of patient-related information from the unit to the municipalities. In the focus groups, it became apparent that HCPs from the municipalities were unaware that these communication lines were nonexistent:

*It’s just as important to send updates to us in the municipality*,* who are out with the patient*,* as it is to send them to the doctor.”* (Nurse)

*I’d love to send one to you as well*,* but we don’t have the option to do that.* (Paramedic)

The theme, *Navigating care through phone calls*, examines how paramedics and other HCPs use continuous phone calls during the task process. Participants highlighted calls made to ensure sufficient information about the patient’s medical history, to initiate treatment, or to arrange follow-up, illustrating how care was actively navigated via the phone.

A paramedic explains the workflow and highlights the large number of calls:

*We have to talk to SO many people. I have to talk to you (ed.: nurse*,* ED)*,* a doctor from the ED*,* a flow coordinator*,* and home care services….* (Paramedic)

Nurses from the municipalities also experienced how missing information generated many calls:

*I ended up calling and talking to four different coordinators across two different hospitals. Unfortunately*,* this is something I’ve noticed several times - that we do not receive the correspondence message.* (Nurse)

The participants who received these phone calls did not describe them as disturbing, but the paramedics highlighted that this might be the case:

*It’s not just me calling all the time and bothering people. Now you’re calling and ‘bothering’ me too – In a good way.* (Paramedic)

Participants perceived that phone calls were necessary in a dynamic and complex acute care service. However, they also agreed that the frequency of calls was not always the most effective way to execute tasks or to navigate care.

#### Relationships

Data on Relationships is outlined in Table 5. Across the three dimensions, eight (7%) of the ties between roles were classified as weak, all in the Shared Knowledge dimension. Specifically, weak ties were reported by physicians from EDs with nurses from municipalities and GPs, by paramedics with GPs, and by the medical dispatchers. Furthermore, medical dispatchers reported weak ties with all roles from organisations outside the prehospital service.

**Table 5:** Relationships dimension of Relational Coordination

Reported by these roles Mean (SD) *N* = 138	Reported with these roles					
	Nurse, ED	Physician, ED	Nurse, Municipality	General Practitioner	Paramedic on PAU	Medical dispatcher
**Shared Goals**						
Nurse, ED	4.5 (1.1)	*4.4 (0.9)*	*4.1 (1.1)*	3.5 (1.1)	*4.6 (1.0)*	*4.5 (0.8)*
Physician, ED	*4.5 (0.8)*	4.5 (0.6)	*3.7 (1.1)*	3.0 (1.0)	*4.6 (0.5)*	*3.9 (1.1)*
Nurse, Municipality	*3.8 (1.2)*	*4.0 (1.0)*	*4.4 (0.8)*	*3.7 (1.0)*	*4.2 (0.8)*	*3.6 (1.1)*
General Practitioner	*4.2 (0.8)*	3.4 (1.3)	4.2 (0.8)	4.5 (0.6)	*4.2 (0.4)*	3.0 (0.8)
Paramedic, PAU	*4.5 (0.5)*	*4.6 (0.6)*	*4.2 (0.8)*	3.3 (1.0)	4.9 (0.3)	3.8 (0.9)
Medical dispatcher	*4.8 (0.4)*	*4.0 (0.9)*	*4.0 (1.0)*	*4.2 (1.2)*	*4.1 (0.9)*	*4.4 (0.7)*
**Shared Knowledge**						
Nurse, ED	4.4 (0.7)	*4.3 (0.6)*	3.5 (0.8)	3.3 (0.8)	*4.5 (0.5)*	3.5 (1.0)
Physician, ED	*4.1 (1.0)*	4.3 (1.0)	**2.9 (1.0)**	**2.7 (1.1)**	4.3 (0.8)	3.6 (1.3)
Nurse, Municipality	3.1 (0.8)	3.3 (0.9)	4.4 (0.9)	3.3 (0.8)	3.2 (0.7)	2.7 (0.8)
General Practitioner	*3.8 (0.4)*	3.0 (1.0)	3.4 (0.9)	4.3 (0.5)	*4.2 (0.4)*	*3.8 (0.5)*
Paramedic, PAU	*3.7 (0.8)*	*4.0 (0.5)*	3.1 (1.0)	**1.8 (0.7**)	*4.7 (0.7)*	**3.4 (1.0)**
Medical dispatcher	**2.9 (0.8)**	**2.9 (0.7)**	**2.8 (0.6)**	**2.6 (0.7)**	4.0 (0.6)	4.6 (0.5)
**Mutual Respect**						
Nurse, ED	*4.8 (0.4)*	*4.7 (0.5)*	*4.1 (1.0)*	*3.8 (0.8)*	*4.7 (0.5)*	*4.4 (0.8)*
Physician, ED	*4.8 (0.4)*	*4.7 (0.6)*	*4.4 (0.8)*	*3.8 (0.9)*	*4.8 (0.4)*	*4.5 (0.7)*
Nurse, Municipality	*4.0 (1.1)*	*4.0 (0.9)*	*4.4 (0.9)*	4.0 (0.9)	*4.1 (0.9)*	*3.8 (1.0)*
General Practitioner	*4.7 (0.5)*	*3.6 (1.5)*	*4.4 (0.5)*	4.5 (0.6)	*4.4 (0.5)*	*4.3 (0.5)*
Paramedic, PAU	*4.7 (0.5)*	*4.6 (0.6)*	*4.4 (0.7)*	*3.6 (0.8)*	*4.9 (0.3)*	*4.4 (0.8)*
Medical dispatcher	*4.5 (0.8)*	*4.1 (0.8)*	*4.0 (0.9)*	*4.3 (0.8)*	*4.1 (0.8)*	4.5 (0.5)

Mean score for the three questions representing the relationships dimension of Relational Coordination within and between roles. Bold values indicate ties classified as weak; italicised values indicate ties classified as strong. Abbreviations: ED, Emergency Department; PAU, Prehospital Assessment Unit; SD, standard deviation

The theme, *Multiple objectives with hindered observers*, describes how the PAU operates within a system in which each organisation and role appears to hold an individual interpretation of the unit’s objective, while possessing detailed insight only into their own segment of the patient care pathway.

The activities of the PAU appear to be shaped by differing objectives set by the various organisations involved. The participants seemed comfortable in their role, aware of the complexity of their service and the large number of HCPs involved in each patient. The units were generally seen as a positive add-on, although they added complexity to the system:

*I think we should see it as a benefit that there are more prehospital services available now than before.* (Physician, ED)

The participants focused on their part of the task process and had limited knowledge of the remaining parts. Several times during the focus groups, participants were surprised by information given by others, leading to participants explaining their part of the pathway to the group:

*It is pretty simple with the unit because the general practitioner doesn’t really have the option to request an activation of it. I mean*,* can they?”* (Physician, ED)

*They can contact the physician at the ED*,* and if they agree that it’s a good idea*,* then they can.* (Paramedic)

During the focus groups, the PAU’s objective and activities were mentioned multiple times. The unit’s success was based on objectives concerning the patients, HCPs, and society. In some examples, the participants stated that the unit meets one objective but not necessarily the others. Concerning the patients visited by the unit, there seems to be agreement about the goal, which is to ensure the best possible care pathway:

*I’ve come to ensure that the patient gets the right treatment*,* at the right place*,* and at the right time* (Paramedic)

The participants considered the benefits and possible improvements of the unit concerning their everyday tasks:

*Well*,* we’re probably at a point where we’d really like you to work around the clock. Because that’s where we have the greatest need. It’s when we have the longest response time from a doctor*,* in the evening and at night.* (Nurse)

The potential benefits can also be assessed from a societal perspective in relation to other acute care services. Alternative care pathways to the units were discussed and show overlapping options in the acute care services:

*I haven’t experienced you taking any blood tests other than CRP. So*,* we’ve kind of had a… a bit of frustration over who does what*,* because the acute team can take down IV fluids*,* and they can also measure a control CRP. It’s just that thing of figuring out what actually makes sense - how the resources are being used.* (Nurse)

An overall objective of the PAU as an alternative to other EMS units was also part of the discussions:

*…from the prehospital perspective*,* that’s also what we’re here for – to relieve the ambulances.* (Paramedic)

These perspectives underscore the necessity of a comprehensive overview of interventions for patients with acute care needs, facilitating shared knowledge and, importantly, establishing a unified objective.

The theme of *Interprofessional tensions in acute care collaboration* builds on the agreement that the hierarchical structure and the intersectoral organisation of the acute care services create regarding roles and responsibilities. However, the participants sometimes questioned whether their current competencies, incentives, roles, and responsibilities were sufficient to achieve the best care pathway.

The participants acknowledged differing levels of competence within the acute care system, although this was not always expressed respectfully:

*We need to provide all information to the technical dispatcher, taking into account that they are not healthcare professionals*. (Nurse)

*… It would be great if you could do that and finish by saying to the technical dispatcher and remember to write down what I just told you” (laughs)* (Paramedic)

Participants shared examples of how economic considerations and limited resources impacted their actions. For example, accepting tasks despite disagreeing with the care pathway chosen by another HCP or when the PAU is activated due to a lack of available ambulances:

*Sometimes the GP makes the patient seem much worse than they actually are*,* to get them admitted. They earn some money and can move on to the next one*,* even though it might not be in the patient’s best interest.* (Nurse)

*It’s the technical dispatcher who*,* has to piece together a puzzle when it comes to managing resources. We’ve had some challenges when we’re dispatched to people sitting out on the street by not having an examination room.* (Paramedic)

Paramedics reported a high degree of trust in the decisions made by ED physicians. However, physicians highlight the complexity of relying on paramedics’ assessments, as they must make decisions - sometimes without detailed knowledge of paramedics’ experience - while bearing the legal responsibility for potentially high-risk choices:

*It’s essential that the person in the unit is experienced. As doctors*,* we really rely on their assessment*,* and that’s the challenge with this system - whether we know each other well enough to trust when the paramedic says something….* (Physician, ED)

*It is an experienced paramedic going out*,* and I often agree with their gut feeling. So*,* whether the patient needs to be admitted or not*,* I trust the assessments made by the experienced prehospital staff.* (Physician, ED)

## Discussion

In this mixed-methods study, we investigated Relational Coordination among healthcare professionals engaged in interdependent task processes associated with the care of patients triaged by the Prehospital Assessment Unit to gain a deeper understanding of the collaborative dynamics within acute care services. Meta-inference, obtained by merging the key quantitative results and qualitative findings [18], is discussed below.

### Relational Coordination

The results and findings in RC confirmed an overall positive collaboration experience within acute care services, despite some ties being classified as weak. Similar findings were reported in a recent study, which also highlighted a generally positive attitude towards PAU [2]. Other studies have reported more contradictory findings. In a study from the United Kingdom about differences in non-conveyance rates, the majority of ambulance services perceived their relationships with key services - such as primary care, community services, and urgent care centres - as poor [5, 24]. Furthermore, the study found that a lack of collaboration can result in patients being conveyed to the ED [24].

### Communication

Communication is recognised as crucial for ensuring integrated care across organisations and between HCPs [25] and was acknowledged as a top priority for patient safety over 20 years ago [26]. The challenges identified in the quantitative dimensions of Frequent Communication and Timely Communication do not explain how to interpret the results. For example, a weak tie in Frequent Communication can be a result of either overly frequent or insufficient communication. The qualitative findings expand on the results by suggesting that these challenges stem from insufficient written communication and too frequent phone calls. Seen in combination, the weak quantitative ties in Frequent Communication and Timely Communication, and the qualitative themes of *Insufficient written communication* and *Navigating care through phone calls* suggest “infrastructure-first implications,” such as standardised task assignments and interoperable correspondence systems across organisational boundaries.

The lack of sufficient information for paramedics in a task assignment documented in this study aligns with experiences reported by paramedics conducting prehospital triage during COVID-19 [3]. The importance of our findings can be interpreted in relation to previous research, suggesting that the written task assignments and access to relevant information impact HCPs early opinion-making, the selection of the most appropriate care pathway, and the non-conveyance decision [27, 28].

The written correspondence messages requested by the municipality nurses in our study have previously been suggested as a facilitator for integrated healthcare and as an alternative to phone calls [29]. Enabling correspondence between PAUs and municipalities could address documented challenges of inadequate written communication and the overreliance on frequent phone calls.

The use of the phone to gather information and explore alternative care pathways in acute care services, as found in our study, has previously been reported in a similar setting [2]. Here, a paramedic referred to a list of relevant phone numbers for collaborating partners as his most important tool [2].

#### Relationships

The HCPs have moderate to strong ties in the dimensions of Shared Goals and Mutual Respect, with a few weak ties in Shared Knowledge. All parties agree on a shared goal: to ensure the best possible care pathway for every patient. However, other findings reveal inconsistencies, with varying descriptions of the unit’s objectives existing, potentially hindering the ability to reach consensus on a shared goal for the patient. Furthermore, our findings suggest that participants question whether the current competencies, incentives, roles, and responsibilities are sufficient to achieve the best care pathway for the patients. The weak ties in Shared Knowledge align with our finding that participants possess limited understanding beyond their specific part of the task process. This convergence suggests that without more explicit role definitions, aligned mandates, and supportive IT systems, informal workarounds risk becoming the default mode of collaboration. As highlighted in studies of organisational boundaries [6] and prehospital collaboration [7], structural supports are required to transform such workarounds into reliable and sustainable care pathways.

Our finding of consensus on an overall common goal aligns with a study on non-conveyance in the United Kingdom, which defines the ultimate objective as “providing each patient with the most appropriate care every time” [24]. The study also identified a need to align objectives and financial incentives around non-conveyance [24]. Research on patients’ perspectives on non-conveyance emphasises the importance of identifying a common goal of care, ideally achieved through joint decision-making involving the patient, their family, and HCPs [30–32]. Differences in perspectives on care pathways have been identified in other areas of inter-organisational collaboration, where hospitals were focused on acute and episodic care, in contrast to holistic, long-term care perspectives from general practice and municipal healthcare [29].

Contrasting viewpoints regarding the professional competencies of paramedics have also been documented in a recent systematic review [1], suggesting the potential to improve Shared Knowledge and Mutual Respect.

Our results regarding the dimension Shared Knowledge align with a study on RC among HCP working at an emergency medical dispatch centre, who experience Shared Knowledge with other nurses in the same role as themselves but lack Shared Knowledge with paramedics working in the EMS, as well as with nurses and medical doctors who call for help [15]. In contrast, a recent study found that paramedics share their experience operating a PAU-like unit with EMS dispatchers, offering learning opportunities for those triaging the patients [2].

### Strengths and limitations

We applied a mixed-methods study design that integrated quantitative data and qualitative insights. This approach enabled a more comprehensive assessment of RC among HCPs, providing a deeper understanding of collaboration within acute care services involving the PAU than any single data source could achieve on its own. Our design added value by contextualising quantitative findings. For example, weak ties in Frequent Communication may reflect either too much or too little interaction, but the RC Survey does not distinguish between the two. Integrating qualitative insights clarified that in this study, weak ties primarily reflected insufficient written communication and reliance on phone calls.

Thes study had a 51.7% survey response rate, and although participation was relatively high, the possibility of non-response bias cannot be excluded. Non-responders may have differed from responders in terms of workload, role, or experience with PAU tasks, which could influence the representativeness of the findings. Another limitation was that recruiting participants for our focus group was highly challenging, resulting in participation from only one GP, one ED nurse, and no medical dispatchers. Absences were due to clinical obligations and reduced medical dispatcher involvement in PAU triage following guideline changes. Their limited participation also reflects that PAU triage is a minor part of their responsibilities. Furthermore, our recruitment of focus group participants primarily from the group that responded to the questionnaire may have constrained the diversity of perspectives captured through the qualitative methods.

Our study aimed to examine and explore RC among HCPs, yet technical dispatchers were omitted despite their role in the task process. This omission can be perceived as a limitation since our theme, *Interprofessional tensions in acute care collaboration* implies that the technical dispatcher’s role and collaboration were somewhat different from those of other HCPs. Similar findings were reported by a recent study from the Central Denmark Region [2].

Finally, while the survey was explicitly designed based on RC theory, the Focus Group Discussion Guide was not, which may have limited the depth of RC-specific reflections; this discrepancy is acknowledged as a methodological limitation.

#### Clinical aspects and future research

This study highlights the critical role of effective collaboration in acute care services for improving care pathways. Future clinical and managerial efforts should focus on developing infrastructure and strategies to improve Communication, clarify roles and responsibilities, and strengthen Mutual Respect among professionals. While the challenges identified might not be evident in all acute care settings, they highlight the importance of examining collaboration during substantial service changes. Additionally, further research into the role of technical dispatchers and their impact on acute care services is warranted.

Previous research has primarily focused on ‘traditional’ ambulance handovers to the ED, emphasising patient safety and positive patient outcomes [33, 34]. Future research on communication practices should focus on developing and implementing standardised methods [26] for all handovers within acute care, including task assignments and correspondence messages to municipalities. Future studies should also investigate how collaboration mechanisms translate into patient outcomes, as these were not measured in the present study.

Finally, future work could explore potential challenges in interpreting RC Survey dimensions, particularly how weak ties may arise from different mechanisms. Such investigations would strengthen the actionable use of this well-established tool while preserving its core strengths.

## Conclusion

In conclusion, while moderate to strong ties and positive experiences in RC, Communication, and Relationships were observed, critical challenges were also identified. Optimising task assignments and facilitating correspondence with municipalities may further enhance care coordination. Additionally, discrepancies in the unit’s objectives and concerns about competencies, incentives, and roles could hinder consensus on patient care pathways. Strengthening Shared Knowledge and aligning perspectives across sectors may further support collaboration. Though situated within the Danish context, these findings resonate with international literature on prehospital and inter-organisational collaboration, reinforcing the universal need for structural supports, interoperable communication systems, and mandate alignment to convert informal workarounds into reliable care pathways. Our study design did not allow causal inference regarding organisational predictors or outcomes of RC, which limits direct implications for practitioners. Future studies could test the hypotheses suggested here by examining outcomes of weaker or stronger RC in PAU and the organisational practices that support it.

## Supplementary Information

Below is the link to the electronic supplementary material.

Supplementary Material 1

## Data Availability

The datasets used and analysed during the current study are available (in anonymised form) from the corresponding author on reasonable request. The Focus Groups Discussion Guide is available as supplementary file 1.

## Abbreviations

ED: Emergency Department
EMS: Emergency medical service
GP: General practitioners
HCP: Healthcare professional
PAU: Prehospital Assessment Unit
RC: Relational Coordination
RTA: Reflexive Thematic Analysis

## References

[1] Blodgett JM, Robertson DJ, Pennington E, Ratcliffe D, Rockwood K. Alternatives to direct emergency department conveyance of ambulance patients: a scoping review of the evidence. Scand J Trauma Resusc Emerg Med. 2021;29(1). 10.1186/s13049-020-00821-x PMC7789540 33407771

[2] Møller FA, Persson ML, Engholm EL, Jensen PHK, Vaeggemose U, Gehrt TB. Is non-conveyance solo-ambulances a useful mean to meet the increasing demand for emergency medical services in Denmark? BMC Health Serv Res. 2025;25(1). 10.1186/s12913-025-12448-8 PMC11852878 40001050

[3] Nielsen VML, Lindskou TA, Weinreich UM, Jespersen MS, Christensen EF, Bøggild H. Decision on non-conveyance of patients suspected of COVID-19 in a novel arrangement with assessment visits by paramedics at home. BMC Emerg Med. 2023;23(1). 10.1186/s12873-023-00826-6 PMC10212731 37237344

[4] Wolthers SA, Blomberg SNF, Breindahl N, Anjum S, Hägi-Pedersen D, Ersbøll A, et al. Association between using a prehospital assessment unit and hospital admission and mortality: a matched cohort study. BMJ Open. 2023;13(9):e075592. 37739475 10.1136/bmjopen-2023-075592 PMC10533654

[5] Knowles E, Bishop-Edwards L, O’Cathain A. Exploring variation in how ambulance services address non-conveyance: a qualitative interview study. BMJ Open. 2018;8(11):e024228. 30498049 10.1136/bmjopen-2018-024228 PMC6278803

[6] Hedqvist AT, Lindberg C, Hagerman H, Svensson A, Ekstedt M. Negotiating care in organizational borderlands: a grounded theory of inter-organizational collaboration in coordination of care. BMC Health Serv Res. 2024;24(1):1438. 39563335 10.1186/s12913-024-11947-4 PMC11577764

[7] Hedqvist AT, Herrera MJ. Ambulance clinicians’ perspectives on interprofessional collaboration in prehospital emergency care for older patients with complex care needs: a mixed-methods study. BMC Geriatr. 2025;25(1):394. 40448003 10.1186/s12877-025-05975-w PMC12124084

[8] Gittell JH. Organizing work to support relational co-ordination. Int J Hum Resource Manage. 2000;11(3):517–39.

[9] Gittell JH, Ali HN. Relational analytics: guidelines for analysis and action. 1st ed. New York, NY: Routledge; 2021.

[10] Bolton R, Logan C, Gittell JH. Revisiting relational coordination: A systematic review. J Appl Behav Sci. 2021;57(3):290–322.

[11] Williams L-MS, Johnson E, Armaignac DL, Nemeth LS, Magwood GS. A mixed methods study of Tele-ICU nursing interventions to prevent failure to rescue of patients in critical care. Telemedicine e-Health. 2019;25(5):369–79. 30036175 10.1089/tmj.2018.0086

[12] Valentine MA, Nembhard IM, Edmondson AC. Measuring teamwork in health care settings: a review of survey instruments. Med Care. 2015;53(4):e16–30. 24189550 10.1097/MLR.0b013e31827feef6

[13] Braun V. Clarke. Thematic analysis: a practical guide. London: Sage; 2022.

[14] Lundstrøm SL, Edwards K, Knudsen TB, Larsen PV, Reventlow S, Søndergaard J. Relational coordination and organisational social capital association with characteristics of general practice. Int J Family Med. 2014;2014:1–7. 10.1155/2014/618435 PMC4089202 25045537

[15] Giachali A. J.M. S. Relational coordination in emergency medical response: the influence of a machine-learning technology improving cardiac arrest identification [Master Thesis]. Copenhagen: Copenhagen Business School; 2018.

[16] House S, Wilmoth M, Stucky C. Relational coordination as a merger and acquisition framework for healthcare organizations. Nurs Manage. 2022;53(2):36–42. 35105844 10.1097/01.NUMA.0000816256.13974.1b

[17] Creswell JWPCV. Designing and conducting mixed methods research. SAGE; 2017.

[18] Fetters MD, Curry LA, Creswell JW. Achieving integration in mixed methods Designs—Principles and practices. Health Serv Res. 2013;48(6pt2):2134–56. 24279835 10.1111/1475-6773.12117 PMC4097839

[19] O’Cathain A, Murphy E, Nicholl J. The quality of mixed methods studies in health services research. J Health Serv Res Policy. 2008;13(2):92–8. 18416914 10.1258/jhsrp.2007.007074

[20] Birk HO, Vrangbaek K, Rudkjobing A, Krasnik A, Eriksen A, Richardson E, et al. Denmark: Health Syst Rev Health Syst Transit. 2024;26(1):1–186. 38841877

[21] Kitzinger J. Focus group research: using group dynamics to explore perceptions, experiences and Understandings. In: Holloway I, editor. Qualitative research in health care. Maidenhead: Open University; 2005. pp. 56–69.

[22] Lundstrøm SL. Relational coordination in Danish general practice. Kgs. Lyngby: Technical University of Denmark; 2014.

[23] World Medical Association. World medical association declaration of helsinki: ethical principles for medical research involving human subjects. JAMA. 2013;310(20):2191–4. 24141714 10.1001/jama.2013.281053

[24] O’Cathain A, Knowles E, Bishop-Edwards L, Coster J, Crum A, Jacques R, et al. Understanding variation in ambulance service non-conveyance rates: a mixed methods study. Health Serv Delivery Res. 2018;6(19):1–192. 29870196

[25] World Health Organization. Continuity and coordination of care: a practice brief to support implementation of the WHO framework on integrated people-centred health services. World Health Organization; 2018.

[26] Joint Commission International, World Health Organization. Patient safety solutions preamble 2007 [Assessed on 07-04-2025]. Available from: https://www.who.int/teams/integrated-health-services/patient-safety/research/patient-safety-solutions

[27] Ebben RHA, Vloet LCM, Speijers RF, Tönjes NW, Loef J, Pelgrim T, et al. A patient-safety and professional perspective on non-conveyance in ambulance care: a systematic review. Scand J Trauma Resusc Emerg Med. 2017;25(1). 10.1186/s13049-017-0409-6 PMC5513207 28716132

[28] Zorab O, Robinson M, Endacott R. Are prehospital treatment or conveyance decisions affected by an ambulance crew’s ability to access a patient’s health information? BMC Emerg Med. 2015;15(1). 10.1186/s12873-015-0054-1 PMC4596371 26446595

[29] Lyngso AM, Godtfredsen NS, Frolich A. Interorganisational integration: healthcare professionals’ perspectives on barriers and facilitators within the Danish healthcare system. Int J Integr Care. 2016;16(1):4. 27616948 10.5334/ijic.2449 PMC5015550

[30] King R, Oprescu F, Lord B, Flanagan B. Patient experience of non-conveyance following emergency ambulance service response: A scoping review of the literature. Australas Emerg Care. 2021;24(3):210–23. 32943367 10.1016/j.auec.2020.08.006

[31] King R, Oprescu FI, Lord B, Flanagan B, Downer T. Patients’ experiences of non-conveyance following an Australian ambulance service paramedic response: A constructivist grounded theory exploration. Paramedicine. 2023;20(3):63–78.

[32] Skaffari E, Iirola T, Nordquist H. Patient experience of non-conveyance in the EMS of Southwest finland: a descriptive survey study. BMC Emerg Med. 2024;24(1):42. 38475735 10.1186/s12873-024-00961-8 PMC10935972

[33] De Lange S, Heyns T, Filmalter C. Clinical practice guidelines for person-centred handover practices in emergency departments: a scoping review. BMJ Open. 2024;14(10):e082677. 39477267 10.1136/bmjopen-2023-082677 PMC11529586

[34] Troyer L, Brady W. Barriers to effective EMS to emergency department information transfer at patient handover: A systematic review. Am J Emerg Med. 2020;38(7):1494–503. 32321683 10.1016/j.ajem.2020.04.036

