# Supplementary material for: Relational coordination among healthcare professionals in acute care: a mixed-methods study of tasks involving prehospital assessment units
Source: BMC Health Serv Res. 2026 Feb 2;26:324. doi: 10.1186/s12913-026-14103-2 (PMC12954915; doi:10.1186/s12913-026-14103-2)
Supplement: Supplementary file 1 — Supplementary Material 1 [file 12913_2026_14103_MOESM1_ESM.pdf]

BMC Health Services Research: **Relational Coordination among Healthcare Professionals in Acute Care: A Mixed-Methods Study of Tasks Involving Prehospital Assessment Units**

Helle Mätzke Rasmussen, Anders Løkke, Peter Biesenbach, Annmarie Lassen, Anne Friesgaard Christensen, Eva Hoffmann, Søren Mikkelsen, Mette Elkjær

Additional File 1: Focus Group Discussion Guide

**Focus Group Discussion Guide**	
Presentation	Each focus group started with a presentation by the moderator, secretary, and the participants.
Experiences	- Would you share your experiences with patients who may need emergency hospitalization?<br>- What experiences do you have with the prehospital assessment unit?<br>- Do you have any specific examples of patient cases where you and the prehospital assessment unit were involved?
Discussion topics	After the presentation of a case, the following topics were discussed:<br>- What are your thoughts on this case?<br>- How well does the overall effort match the patient's needs?<br>- How do the prehospital assessment unit's capabilities for examination, intervention, and referral align with the patient's needs?<br>- What alternative options would you consider for the patient?
Wrap-up	- Anything you would like to add?<br>- Is there something you feel we haven't explored in enough depth?

**Translated and shortened case presentations**	
**Case 1**<br>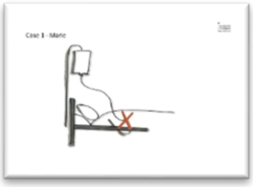	Marie, an 85-year-old woman with dementia, is on respite care and receiving intravenous treatment. After being discharged two days ago, she removed her IV, and the nurse couldn't reinsert it, prompting a call to the paramedic on the Prehospital Assessment Unit. The paramedic successfully placed a new IV and informed the Municipal Acute Care Team, determining there was no need for further contact with the Emergency Department or General Practitioner.

**Translated and shortened case presentations** (continued)	
**Case 2**<br>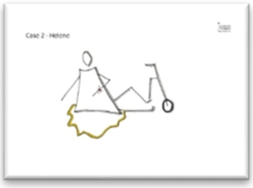	Helene, an 82-year-old woman, lives alone with help from home care and her family. A visit at her General Practitioner revealed slightly elevated infection markers, the doctor recommended she go to the Emergency Department. However, the department suggested she be evaluated by the Prehospital Assessment Unit.<br>When the paramedic arrived, he found Helene on the floor with a minor arm injury from a fall. She mentioned she had been sleeping for four days but didn't feel any pain. The paramedic decided that home care wasn't enough and called an ambulance to take her to the Emergency Department.
**Case 3**<br>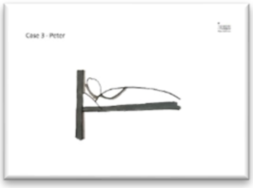	Peter, a 93-year-old man, lives in a residential care home and has had diarrhea, nausea, and stomach pain for the past day. The staff, concerned, contacted his General Practitioner who suggested an assessment by the Prehospital Assessment Unit.<br>The paramedic's assessment showed normal breathing, warm dry skin, regular pulse, and no edema. Peter was alert and oriented, reporting his usual mild leg pain. A blood test indicated slight dehydration. After consulting with the Medical Doctor at the Emergency Department, intravenous fluid treatment was initiated. The Municipal Acute Care Team was informed, and both Peter and the staff felt reassured. An update was sent to his General Practitioner.
**Case 4**<br>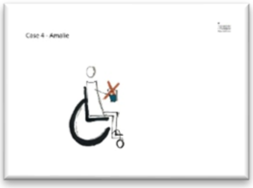	Amalie, 54, has multiple sclerosis, uses a wheelchair, and needs daily help. She had three visits from the Prehospital Assessment Unit in one month:<br>1) Her husband called the Emergency Medical Dispatch Centre, who activated the unit. The paramedic examined her and found high infection markers, low oxygen, and low blood pressure. She was conveyed by ambulance and admitted to the Emergency Department for pneumonia treatment.<br>2) After contact to her General Practitioner, she was examined again. She refused to go to the Emergency Department, so treatment with antibiotics and intravenous fluids was started at home.<br>3) After contact to the out-of-hours General Practitioner, who asked to have her admitted at the emergency Department, the unit traiged her and initiated treatment with intravenous fluid at home.
